# Association of atrial myopathy in mitral valve disease on safety outcomes in left atrial appendage closure

**DOI:** 10.1007/s00392-022-02151-7

**Published:** 2023-02-05

**Authors:** Shinwan Kany, Johanna Skibowski, Claus-Heinrich Müller, Volker Geist, Jörn Schmitt, Feraydoon Niroomand, Birgit Hailer, Sven Pleger, Ibrahim Akin, Matthias Hochadel, Jochen Senges, Edith Lubos

**Affiliations:** 1 Division of Cardiology, University Heart and Vascular Center Hamburg-Eppendorf, Martinistraße 52, 20251 Hamburg, Germany; 2 Division of Cardiology and Intensive Care, Klinikum Lüneburg, Lüneburg, Germany; 3 Division of Cardiology, Segeberger Kliniken, Bad Segeberg, Germany; 4 Division of Cardiology, University Hospital Giessen, Giessen, Germany; 5 Division of Cardiology, St. Josefskrankenhaus Heidelberg, Heidelberg, Germany; 6 Division of Cardiology and Angiology, Phillipusstift Essen, Essen, Germany; 7 Division of Cardiology, University Hospital Heilberg, Heidelberg, Germany; 8 Division of Cardiology, University Hospital Mannheim, Mannheim, Germany; 9 Stiftung Für Herzinfarktforschung, Ludwigshafen, Germany; 10 Division of Cardiology, Marienkrankenhaus Hamburg, Hamburg, Germany

**Keywords:** Atrial fibrillation, Mitral valve regurgitation, Safety outcomes, Left atrial appendage occlusion

## Abstract

**Background:**

Patients undergoing left atrial appendage (LAA) occlusion (LAAO) are multi-morbid, including mitral valve disease (MVD) which is associated with anatomic changes of the left atrium (LA). This study aims to identify how atrial myopathy in MVD influences outcomes in LAAO.

**Methods:**

Atrial myopathy in MVD was defined as LA diameter > 45 mm (♀) and > 48 mm (♂) and existing MVD or history of surgical/interventional treatment. Patients were compared with controls from the prospective, multicentre LAArge registry of LAAO.

**Results:**

A total of 528 patients (52 MVD, 476 no-MVD) were included. The MVD group was significantly more likely to be older (78.2 years vs 75.9 years, *p* = 0.036) and female (59.6% vs 37.8%, *p* = 0.002). Altered LA anatomy was observed in MVD with significantly larger LA diameter (53 mm vs. 48 mm, *p* < 0.001) and LAA Ostia [at 135° 23.0 mm (20.5, 26.0) vs 20.0 mm (18.0, 23.0), *p* = 0.002]. Implant success was high with 96.2% and 97.9%, respectively, without differences in severe complications (7.7% vs 4.6%, *p* = 0.31). One-year mortality (17.8% vs 11.5%, *p* = 0.19) and a combined outcome of death, stroke, and systemic embolism (20.3% vs 12.4%, *p* = 0.13) were not different. Independent predictors of the combined outcome were peripheral artery disease (HR 2.41, 95% CI 1.46–3.98, *p* < 0.001) and chronic kidney disease (HR 3.46, 95% CI 2.02–5.93, *p* < 0.001) but not MVD and atrial myopathy.

**Conclusion:**

Patients with MVD present with altered LA anatomy with increased LA and LAA diameter. However, procedural success and safety in LAAO are not compromised. One-year mortality is numerically higher in patients with MVD but driven by comorbidities.

**Graphical abstract:**

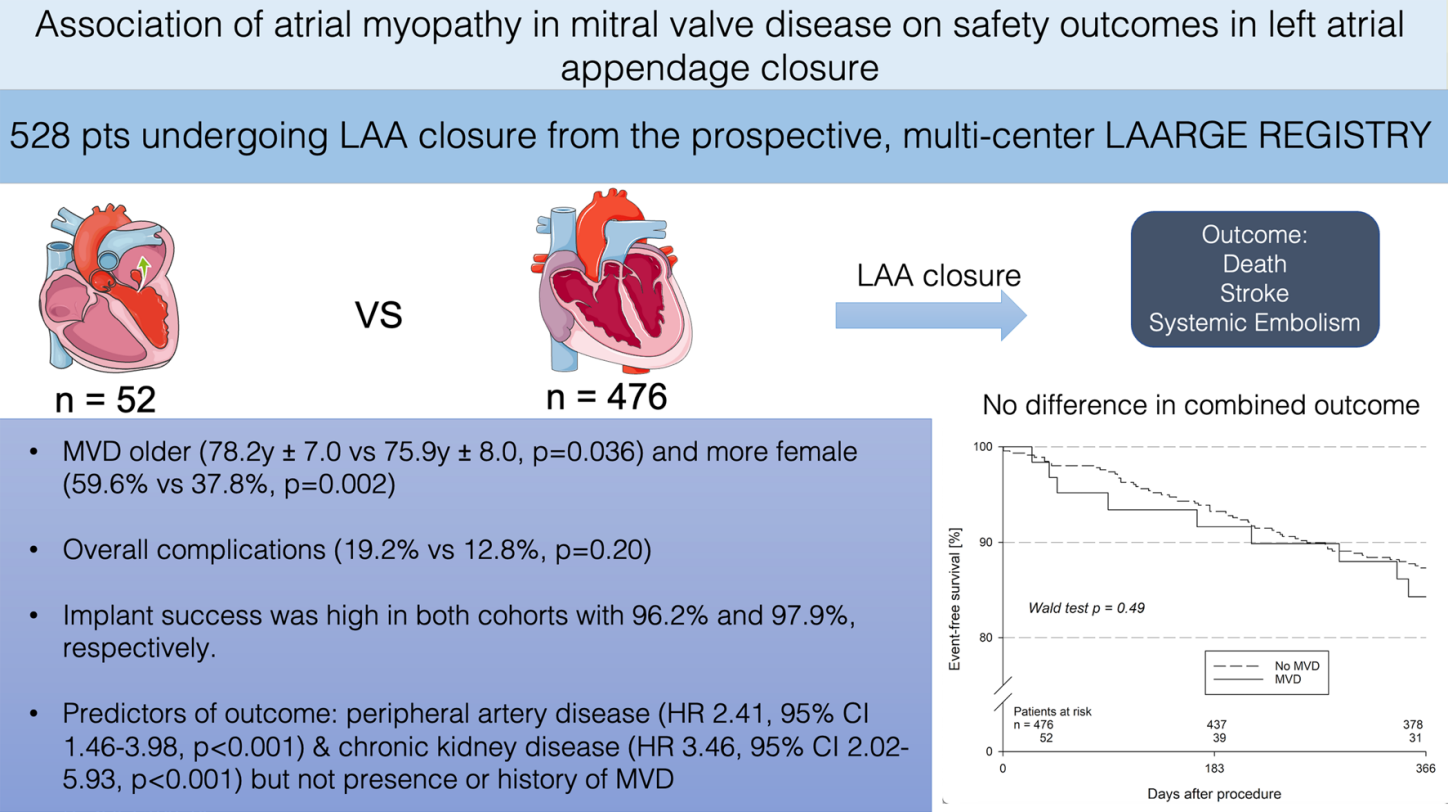

**Supplementary Information:**

The online version contains supplementary material available at 10.1007/s00392-022-02151-7.

## Introduction

Atrial fibrillation (AF) is the most common arrhythmia affecting more than 33 million people worldwide in 2010 [1]. AF is associated with an increase in stroke risk and mortality and its prevalence is rising [2]. While vitamin K antagonists such as Warfarin and non-vitamin K oral anticoagulants (NOAC) reduce stroke and mortality in AF, they substantially increase the risk for major bleedings [3]. In AF patients with a high risk for stroke and high risk for bleeding and/or contraindication to OAC, left atrial appendage occlusion (LAAO) may address both issues [4]. The five-year outcome data of the randomized controlled trials PROTECT-AF (WATCHMAN Left Atrial Appendage System for Embolic Protection in Patients With Atrial Fibrillation) and PREVAIL-AF (Evaluation of the WATCHMAN LAA Closure Device in Patients With Atrial Fibrillation Versus Long Term Warfarin Therapy) support a non-inferiority of LAAO to OAC with a significant decrease in hemorrhagic stroke [5]. However, comorbidities such as heart failure and chronic kidney disease impair clinical outcomes in patients with AF [6]. In fact, in patients undergoing transcatheter edge-to-edge repair (TEER), AF is associated with more bleeding, HF hospitalization, and mortality [7]. The mitral valve is of particular interest in LAAO because of the anatomical proximity and the effects on left atrial anatomy. Left atrial size is a predictor of cardiac death in heart failure before [8].

To our knowledge, this remains a field with no to little evidence, even though in a single-center study of 122 TEER patients, around 50% would also qualify for LAAO [9]. Currently, the first studies are evaluating a combined approach of both procedures even when no data on outcomes on LAAO in patients with mitral disease is available [10].

The German LAArge registry is an independently financed prospective, non-randomized registry of patients undergoing LAAO.

In this study, we aim to investigate the influence atrial myopathy due to mitral valve disease may have on in-hospital and long-term safety outcomes in patients after LAAO.

## Methods

### Data collection and LAArge registry

The non-profit organization “Institut für Herzinfarktforschung” (IHF, Ludwigshafen, Germany) manage and oversee the multicenter German left atrial appendage occlusion registry (LAArge). Thirty-eight centers participated in this prospective, non-randomized study. No funding from industry sources was used for the project. From all enrolled patients, written informed consent was obtained. The privacy measures and data collection have been described previously [11]. Using a web-based electronic case report form, baseline characteristics, procedural data as well as in-hospital data were collected and checked for plausibility. All transmitted data were encrypted and stored on servers maintained by IHF. Echocardiographic follow-up was documented at the standard follow-up at each participating site after LAAO, usually 3–6 months. The IHF conducted the 1-year follow-up by reports from the implanting center and via a standardized phone interview. The study was carried out according to the declaration of Helsinki and approved by the ethics committee of the State Chamber of Medicine in Rhineland-Palatinate, Germany.

### Mitral valve disease definition and procedural methods

Mitral valve disease (MVD) was defined as pre-existing mitral regurgitation and/or TEER or surgical MV repair (SMVR) combined with a left atrial (LA) diameter of > 45 mm in women and > 48 mm in men. The reasoning was to include any irregular mitral valve anatomy, due to pathological MR or surgically or percutaneously treated MV. In addition, grading of mitral regurgitation after TEER becomes increasingly difficult. Patients were either allocated to the MVD group or the no-MVD group if the criteria were met. The detailed procedural methods have been described previously [11]. Patients were screened and enrolled following current guidelines and best medical practice [12]. Generally, AF patients with significant stroke risk and contraindication to anticoagulation were included. Device selection and pre-procedural imaging were left to the operator’s preference. The antithrombotic regime and procedural protocol were conducted according to the implanting center. Transesophageal echocardiography (TEE) was carried out to exclude patients with intracardiac thrombus and define anatomy for technical feasibility. Procedures were carried out in light sedation using propofol or general anesthesia.

### Outcomes

In-hospital data included serious adverse events such as device embolization, peri-device leak, bleeding, stroke, and groin complications among others. Predefined safety outcomes are given in supplemental Table 1. Implantation success and adverse device events were defined as given in the Munich consensus document [13]. The primary outcome was a combined outcome of death, stroke, and systemic embolism after a year. Adverse events were collected for in-hospital events and follow-up events. Routine TEE follow-up was carried out but not mandatory to report as many implanting centers may have referred the patients to local centers for TEE follow-up.

### Statistical analysis

Normally distributed continuous data are given as means ± of the standard deviation (SD), otherwise shown as medians with interquartile ranges (25th and 75th percentiles). Categorical data are presented in relative percentage and absolute values. Fisher’s exact test was used for rates of in-hospital complications. Statistical differences between both groups were compared using either a Chi-square test or the Mann–Whitney–Wilcoxon test. The 12-month event rates of death, the composite outcome of death and stroke, and a composite of death, stroke, and systemic embolism were calculated by the Kaplan–Meier method. The outcomes were compared between age groups using the log-rank test. Hazard ratios (HR) with 95% confidence intervals (CI) were estimated using Cox regression without adjustment. All statistical comparisons were two-sided, and *p* values < 0.05 were considered statistically significant. Analyses were performed using the Statistical Analysis System (SAS, Version 9.4, SAS Institute Inc., Cary, NC, USA).

## Results

### Baseline characteristics

A total of 555 patients were included in this study. The MVD cohort included 52 Patients and the no-MVD cohort 476 patients (Table 1). The MVD group was significantly more likely to be older (78.2 years ± 7.0 vs 75.9 years ± 8.0, *p* = 0.036) and female (59.6% vs 37.8%, *p* = 0.002). In the MVD cohort, 5.8% had a history of TEER and 11.5% had a history of SMVR. Cardiomyopathy (21.2% vs. 5.9%, *p* < 0.001) and chronic kidney disease (59.6% vs 36.8%, *p* = 0.001) were significantly higher in the MVD group. CHA_2_DS_2_-VASc (5.1 ± 1.5 vs. 4.5 ± 1.5, *p* = 0.009) and HAS-BLED-Score (4.4 ± 1.1 vs. 3.8 ± 1.1, *p* = 0.003) were significantly higher in MVD patients compared with the no-MVD group.

**Table 1:** Baseline characteristics

	MVD cohort (52)	No-MVD cohort (476)	*p *value
Age (years)	78.2 ± 7.0	75.9 ± 8.0	0.036
Female	59.6% (31)	37.8% (180)	0.002
Height (cm)	166 (158, 170)	172 (164, 176)	< 0.001
Weight (kg)	68 (63, 83)	80 (70, 89)	< 0.001
Mitral valve disease	100.0% (52)	0% (0)	–
History of TEER	5.8% (3)	–	–
History of SMVR	11.5% (6)	–	–
Atrial fibrillation			
Paroxysmal	38.5% (20)	40.5% (193)	0.77
Persistent	19.2% (10)	17.9% (85)	0.81
Permanent	42.3% (22)	41.6% (198)	0.92
Coronary artery disease	46.2% (24)	46.4% (221)	0.97
History of MI	7.7% (4)	9.9% (47)	0.61
Cardiomyopathy	21.2% (11)	5.9% (28)	< 0.001
Hypertrophic cardiomyopathy	0% (0)	0.4% (2)	0.64
Dilated cardiomyopathy	7.7% (4)	4.0% (19)	0.21
Other cardiomyopathy	13.5% (7)	1.5% (7)	< 0.001
Congestive heart failure	34.6% (18)	28.4% (135)	0.35
LVEF (%)	54 (50, 60)	60 (50, 60)	0.077
Hypertensive cardiomyopathy	13.5% (7)	28.8% (137)	0.019
Anemia	34.6% (18)	21.6% (103)	0.035
Extracardiac history			
Diabetes mellitus	42.3% (22)	33.6% (160)	0.21
Chronic kidney disease	59.6% (31)	36.8% (175)	0.001
Vascular disease (e.g., PAD)	36.5% (19)	24.8% (118)	0.066
History of stroke	21.2% (11)	21.7% (103)	0.93
Chronic liver disease	11.5% (6)	9.2% (44)	0.59
Alcohol use disorder	0.0% (0)	4.0% (19)	0.14
Risk scores			
CHA_2_DS_2_-VASc Score	5.1 ± 1.5	4.5 ± 1.5	0.009
CHA_2_DS_2_-VASc Score > 2	94.2% (49)	90.8% (432)	0.40
HAS-BLED Score	4.4 ± 1.1, *N* = 52	3.8 ± 1.1, *N* = 497	0.003

Displayed are percentage and numbers or median and quartiles; *p *values < 0.05 are considered significant, tested with either Pearson chi-squared test or Mann–Whitney–Wilcoxon test

*MVD* mitral valve disease, *TMVR* transcatheter mitral valve repair, *SMVR* surgical mitral valve repair, surgical aortic valve replacement, *MI* myocardial infarction, *LVEF* left ventricular ejection fraction, *PAD* peripheral artery disease

### Left atrial appendage anatomy

Data for the left atrial appendage anatomy are presented in Table 2. The MVD cohort presented with significantly larger LA diameter (53 mm vs. 48 mm, *p* < 0.001) as well as larger LAA ostia (at 135° 23.0 mm (20.5, 26.0) vs 20.0 mm (18.0, 23.0), *p* = 0.002). There were no significant differences in LA sludge or LAA thrombus formation. The chicken wing morphology occurred the most in both groups (34.6% vs 46.6%, *p* = 0.10), followed by the windsock (13.5% vs 16.2%, *p* = 0.61) and cauliflower (17.3% vs 15.5%, *p* = 0.74) morphology and less often by the cactus (5.8% vs 9.9%, *p* = 0.33) morphology. Patients with MVD were more likely to have an atypical (classification not applicable) LAA morphology (28.8% vs 11.7%, *p* < 0.001).

**Table 2:** Left atrial appendage anatomy

	MVD cohort (52)	No-MVD cohort (476)	*p *value	OR (95% CI)
LA diameter (mm)	53 (50, 58)	48 (44, 51)	< 0.001	
LAA thrombus formation				
LAA thrombus	0.0% (0/52)	0.9% (4/459)	0.50	
LAA sludge	21.2% (11/52)	14.9% (67/451)	0.23	1.54 (0.75–3.14)
LAA morphology				
Cactus	5.8% (3/52)	9.9% (44/444)	0.33	0.56 (0.17–1.86)
Chicken wing	34.6% (18/52)	46.6% (207/444)	0.100	0.61 (0.33–1.11)
Windsock	13.5% (7/52)	16.2% (72/444)	0.61	0.80 (0.35–1.85)
Cauliflower	17.3% (9/52)	15.5% (69/444)	0.74	1.14 (0.53–2.44)
Not applicable	28.8% (15/52)	11.7% (52/444)	< 0.001	3.06 (1.57–5.95)
No. of lobi				
1 Lobus	53.8% (28/52)	54.0% (238/441)		
2 Lobi	44.2% (23/52)	37.9% (167/441)		
> 2 Lobi	1.9% (1/52)	8.2% (36/441)		
LAA dimensions				
Dimension ostium measured (at least 1 plane)	80.8% (42/52)	85.8% (381/444)	0.33	0.69 (0.33–1.45)
Ostium 0° (mm)	22.0 (21.0, 23.0)	20.0 (18.0, 22.0)	0.001	
Ostium 45° (mm)	23.0 (20.0, 25.0)	20.0 (17.0, 22.0)	< 0.001	
Ostium 90° (mm)	21.0 (19.0, 25.0)	20.0 (17.0, 22.0)	0.055	
Ostium 135° (mm)	23.0 (20.5, 26.0)	20.0 (18.0, 23.0)	0.002	

Displayed are percentage and numbers or median and quartiles; *p *values < 0.05 are considered significant, tested with either Pearson Chi-squared test or Mann–Whitney–Wilcoxon test

*MVD* mitral valve disease, *OR* odds ratio, *CI* confidence interval, *LA* left atrium, *LAA* left atrial appendage

### Procedural data

Implant success was high in both cohorts with 96.2% and 97.9%, respectively. There was a significant difference in subpar device position in the MVD group [3.8% (2/52) vs 0.2% (1/476), *p* < 0.001] compared with the no-MVD cohort. No difference in device selection was reported. The Watchman device was the most common device (50% and 42.0%), followed by the Amplatzer Cardiac Plug (19.2% and 29.8%) and the Amplatzer Amulet (25.0% and 25.6%). Device embolization occurred in 1/52 procedures in the MVD cohort and 6/476 cases in the no-MVD cohort (*p* = 0.69). Peri-device leak and left–right shunt occurred in both groups without significant differences. No peri-device leaks > 5 mm were observed in either cohort (Table 3).

**Table 3:** Procedural data

	MVD cohort (52)	No-MVD cohort (476)	*p *value	OR (95% CI)
Implant success	96.2% (50)	97.9% (466)	0.42	0.54 (0.11–2.52)
Subpar device position	3.8% (2)	0.2% (1)	< 0.001	19.00 (1.69–213.26)
Anesthesia				
Conscious sedation	86.5% (45/52)	85.5% (406/475)	0.75	1.15 (0.50–2.64)
General anesthesia	11.5% (6/52)	12.2% (58/475)	0.83	0.91 (0.37–2.22)
LAAO device				
Watchman	50.0% (26)	42.0% (200)	0.27	1.38 (0.78–2.45)
Amplatzer Cardiac Plug	19.2% (10)	29.8% (142)	0.11	0.56 (0.27–1.15)
Amplatzer Amulet	25.0% (13)	25.6% (122)	0.92	0.97 (0.50–1.87)
Other device*	5.8% (3)	2.5% (12)	0.18	2.37 (0.65–8.68)
Periprocedural data				
Sheath retractions	1.5 ± 1.1, *N* = 52	1.6 ± 1.2, *N* = 460	0.49	
Duration (min)	62 (54, 86)	57 (43, 76)	0.054	
Fluoroscopy duration	11 (8, 15)	10 (7, 15)	0.18	
Dose area product (cGy cm^2^)	2272 (904, 3400)	2086 (792, 4368)	0.74	
Device embolization	1.9% (1/52)	1.3% (6/476)	0.69	1.54 (0.18–13.01)
Catheter-based retraction	100.0% (1/1)	100.0% (6/6)	–	–
Surgical salvage	0.0% (0/1)	0.0% (0/6)	–	–
Peri-device leak	5.8% (3/52)	5.3% (24/455)	0.88	1.10 (0.32–3.78)
< 3 mm	33.3% (1/3)	79.2% (19/24)		0.13 (0.01–1.76)
3–5 mm	66.7% (2/3)	20.8% (5/24)		7.60 (0.57–101.79)
> 5 mm	0.0% (0/3)	0.0% (0/24)		–
Left–right shunt	7.7% (4/52)	4.9% (23/467)	0.39	1.61 (0.53–4.85)

Displayed are percentage and numbers or median and quartiles

*MVD* mitral valve disease, *OR* odds ratio, *CI* confidence interval, *LAAO* left atrial appendage occlusion

*Other devices include Occlutech, LAmbre and LARIAT; *p *values < 0.05 are considered significant, tested with Fisher’s exact test

### In-hospital safety data

The data for in-hospital safety are presented in Table 4. The MVD cohort had a longer hospital stay after the procedure compared with the no-MVD cohort [3 days (2, 6) vs 2 days (2, 3), *p* = 0.001]. There were no MACCE (death, stroke, or myocardial infarction) in the MVD cohort and 3 events in the no-MVD cohort. Other severe complications occurred in the MVD group with 5.8% and with 2.9% in the no-MVD cohort (*p* = 0.23). These included pericardial effusion with need for interventional drainage (3.58% vs 2.10%, *p* = 0.33), AV-Fistula (1.9% vs. 0.84%, *p* = 0.11), and severe bleedings (3.8% vs. 0.84%, *p* = 0.11) without significant differences between both groups. Moreover, moderate complications (13.5% vs 9.9%, *p* = 0.47) and overall complications (19.2% vs 12.8%, *p* = 0.20) were comparable between both cohorts.

**Table 4:** In-hospital safety data

	MVD cohort (52)	No-MVD cohort (476)	*p *value	OR (95% CI)
Hospital days after procedure	3 (2, 6)	2 (2, 3)	0.001	–
MACCE (death, MI, stroke)	0.0% (0)	0.63% (3)	1.00	–
Death	0.0% (0)	0.42% (2)	1.00	–
Myocardial infarction	0.0% (0)	0.21% (1)	1.00	–
Stroke	0.0% (0)	0.21% (1)	1.00	–
Other severe complications	5.8% (3)	2.9% (14)	0.23	2.02 (0.56–7.28)
Severe bleeding	3.8% (2)	0.8% (4)	0.11	4.72 (0.84–26.42)
AV-Fistula/pseudoaneurysm	1.9% (1)	0.84% (4)	0.41	2.31 (0.25–21.10)
Pericardial effusion—surgical treatment	0.0% (0)	0.21% (1)	1.00	–
Pericardial effusion—interventional treatment	3.58% (2)	2.10% (10)	0.33	1.86 (0.40–8.75)
Hemo-/Pneumothorax—surgical treatment	0.0%(0)	0.0% (0)	–	–
Device embolization—surgical treatment	0.0% (0)	0.0% (0)	–	–
Device embolization—interventional treatment	0.0% (0)	0.4% (2)	1.00	–
MACCE + other severe complication	7.7% (4)	4.6% (22)	0.31	1.72 (0.57–5.20)
Moderate complications	13.5% (7)	9.9% (47)	0.47	1.42 (0.61–3.33)
Minor complications	5.77% (3)	2.94% (14)	0.23	2.02 (0.56–7.28)

Severe bleeding included bleeding included hemodynamic instability, the need of transfusion, retroperitoneal or intracranial bleedings; displayed are percentage and numbers or median and quartiles; *p *values < 0.05 are considered significant, tested with Fisher’s exact test

*MVD* mitral valve disease, *OR* odds ratio, *CI* confidence interval, *MI* myocardial infarction, *AV* arteriovenous, *TIA* transient ischemic attack, *CPR* cardiopulmonary resuscitation

### Follow-up safety data

**Fig. 1:**
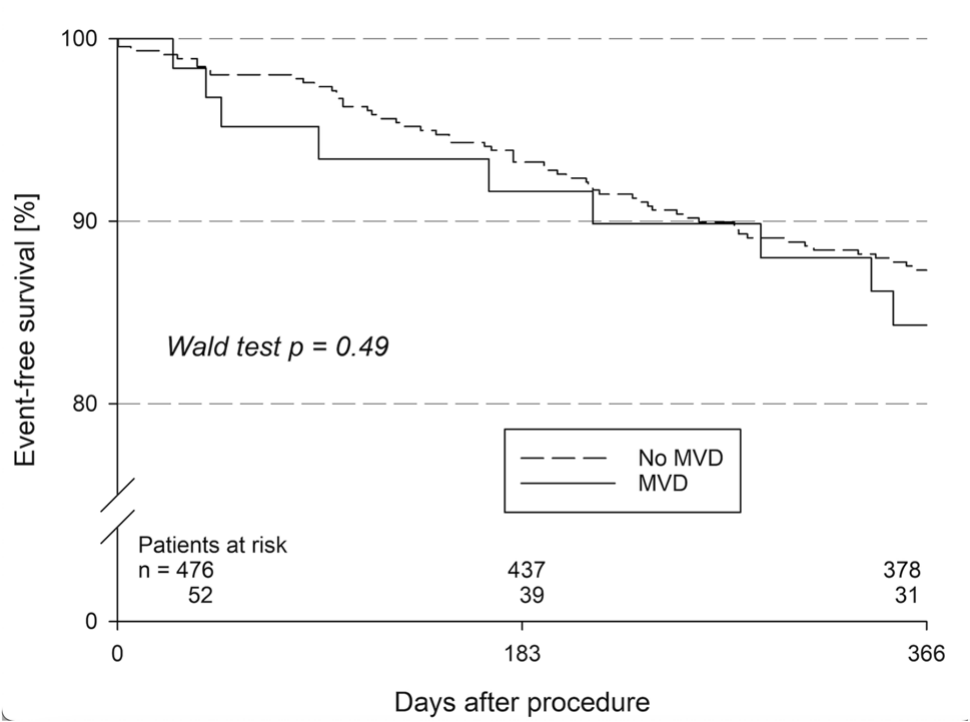
Kaplan–Meyer estimates of event-free survival (death, stroke, systemic embolism) in patients with the presence of mitral valve disease (MVD) or not (no MVD). *p *values < 0.05 considered significant

The Follow-up data are shown in Table 5. The follow-up rate was high in both groups with 92.3% in the MVD cohort and 98.7% in the no-MVD cohort. Device embolization was comparable in both groups (1.9% vs. 2.3%, *p* = 0.86). There were no differences in major complications including stroke, myocardial infarction, and moderate or severe bleeding. Follow-up echocardiography data were obtained in 42.3% and 31.9% of the patient at a mean follow-up duration of 163 and 97 days. No differences were observed in LA thrombi (4.5% vs 6.0%, *p* = 1.00) or overall peri-device leak (27.3% vs 16.6%, *p* = 0.24). Leaks over 5 mm were not observed in either cohort. One-year mortality was numerically higher in the MVD cohort (17.8% vs 11.5%, *p* = 0.19) as well as a combined outcome of death, stroke, and systemic embolism (20.3% vs 12.4%, *p* = 0.13) without reaching statistical significance (Fig. 1). Significant predictors of the combined outcome after adjusting were peripheral artery disease (HR 2.41, 95% CI 1.46–3.98, *p* < 0.001) and chronic kidney disease (HR 3.46, 95% CI 2.02–5.93, *p* < 0.001) but not the presence or history of MVD (Table 6). Further data on safety outcomes (Table S1) and antithrombotic therapy (Table S2) are provided in the supplement.

**Table 5:** Follow-up safety data

	MVD cohort (52)	No-MVD cohort (476)	*p *value	OR (95% CI)
FU documented	92.3% (48/52)	98.7% (470/476)		
Procedure ⟶ FU-contact (days)	374 (366, 403)	381 (368, 408)	0.12	
Device embolization	1.9% (1/52)	2.3% (11/475)	0.86	0.83 (0.10–6.54)
Surgical treatment	(0/0)	18.2% (2/11)	0.64	–
Interventional treatment	100% (1/1)	63.6% (7/11)	0.46	–
Conservative treatment	(0/0)	18.2% (2/11)	0.64	–
Groin complications	1.9% (1/52)	4% (19/475)	0.46	0.47 (0.06–3.59)
Surgical treatment	0.0% (0/1)	15.8% (3/19)	0.67	–
Blood transfusion	0.0% (0/0)	0.0% (0/19)	–	–
Conservative treatment	100% (1/1)	84.2% (16/19)	0.67	–
Pericardial effusion	9.6% (5/52)	4.4% (21/475)	0.10	2.30 (0.83–6.38)
Surgical treatment	0.0% (0/5)	5.0% (1/20)	0.61	–
Interventional treatment	40.0% (2/5)	55.0% (11/20)	0.55	–
Conservative treatment	60% (3/5)	40% (8/20)	0.42	–
Echo-FU documented	42.3% (22/52)	31.9% (151/474)	0.16	
Procedure ⟶ Echo-FU-contact (days)	163 (77, 223)	95 (48, 178)	0.12	
Peri-device leak	27.3% (6/22)	16.6% (25/151)	0.24	1.89 (0.67–5.30)
< 3 mm	66.7% (4/6)	84.0% (21/25)	0.57	0.38 (0.05–2.83)
3–5 mm	33.3% (2/6)	16.0% (4/25)	0.57	2.63 (0.35–19.51)
> 5 mm	0.0% (0/6)	0.0% (0/25)	–	–
LA thrombus	4.5% (1/22)	6.0% (9/150)	1.00	0.75 (0.09–6.19)
Stroke	2.8% (1/36)	1.0% (4/389)	0.36	2.75 (0.30–25.28)
TIA	0.0% (0/36)	0.5% (2/389)	0.67	–
Myocardial infarction	2.8% (1/36)	0.8% (3/389)	0.30	3.68 (0.37–36.28)
Bleeding (severe or moderate)	7.7% (4/52)	6.1% (29/475)	0.55	1.28 (0.43–3.80)
Severe bleeding	3.8% (2/52)	2.3% (11/475)	0.37	1.69 (0.36–7.83)
Moderate bleeding	3.8% (2/52)	3.8% (18/475)	1.00	1.02 (0.23–4.51)
Composite outcomes				
Mortality	17.8% KM	11.5% KM	0.19LO	1.64 (0.78–3.45) HR
Death/stroke	20.3% KM	12.2% KM	0.12LO	1.75 (0.86–3.53) HR
Death/stroke/SE	20.3% KM	12.4% KM	0.13LO	1.71 (0.85–3.45) HR

Displayed are percentage and numbers or median and quartiles; *p *values < 0.05 are considered significant, tested with either Pearson chi-squared test or Mann–Whitney–Wilcoxon test

*MVD* mitral valve disease, *OR* odds ratio, *CI* confidence interval, *FU* follow-up, *TIA* transient ischemic attack, *SE* systemic embolism, *KM* Kaplan–Meier estimate, *LO* Log-rank test, *HR* hazard ratio

**Table 6:** Adjusted cox regression for predictors of death, stroke, systemic embolism (67/528) in patients undergoing left atrial appendage occlusion

Variable	Adjusted hazard ratio	95% CI	*p *value
Mitral valve disease	1.31	0.61–2.80	0.495
Age (per 10-year increase)	1.29	0.91–1.83	0.150
Female sex	0.72	0.41–1.24	0.234
Cardiomyopathy	0.83	0.34–2.03	0.682
Peripheral arterial disease	2.41	1.46–3.98	< 0.001
History of chronic kidney disease	3.46	2.02–5.93	< 0.001
Anemia	0.99	0.58–1.70	0.979

*p *values < 0.05 are considered significant, tested with either Pearson Chi-squared test or Mann–Whitney–Wilcoxon test

*HR* hazard ratio

## Discussion

### Main findings

Patients with atrial myopathy and mitral valve disease referred for LAAO have more comorbidities than patients without a history of mitral valve disease. Even though larger LA and LAA orifices are observed, procedure success is high and comparable to patients without MVD. Cardiovascular events after one year are more common in patients with mitral valve disease but are driven by comorbidities.

### Clinical characteristics and observed events

In patients with mitral valve disease, the occurrence of new-onset atrial fibrillation is high, reaching over 48% at a 10-year follow-up, and is associated with increased cardiac mobility [14]. In a more recent cohort of 2425 patients with degenerative mitral regurgitation, AF was associated with increased overall mortality [15]. In our study, MVD in a cohort of patients with atrial fibrillation is associated with numerically higher mortality. Both conditions are expected to rise with an aging population and increasing comorbidity.

We did not find that the presence of MVD and atrial myopathy is a predictor of cardiovascular events after LAAO. In line with previous data, comorbidities such as chronic kidney disease are stronger factors for mortality in AF [16]. These results are an important safety signal for patients with MVD as LAAO can be conducted in these patients without additional risk to the basic risk from comorbidities.

Another shared trait of both conditions is atrial myopathy with an increased size of both LA and LAA. In fact, in a prospective imaging study using both computed tomography and echocardiography, the most significant determinants of LA enlargement were AF and mitral regurgitation [17]. In our study, atypical morphology of the LAA was observed at a higher rate in those with MVD and atrial myopathy. LAA remodeling has been described in patients with persistent AF [18]. While this has not been reported in MVD, a higher volume load may lead to further remodeling.

However, it must be noted that atrial myopathy is a complex trait that is reflected in electrocardiograms, magnetic resonance imaging, strain imaging, and invasive electrophysiologic studies [19]. In the absence of this data, we chose the combination of LA enlargement and mitral valve disease in atrial fibrillation based on a study that reported that MR in HFpEF reflected LA myopathy even in the absence of AF [20]. Further studies have shown that the combination of mitral valve disease, atrial fibrillation, and left atrial enlargement is highly associated with LA myopathy [21].

Yet, implant success was high in both groups without a significant difference (96.2% vs. 97.9%). The complex anatomy and larger LAA observed with MVD and atrial myopathy are not associated with longer procedures or enhanced support with general anesthesia. While we observed a higher incidence of subpar device position with MVD, event rates are small and should be carefully considered. Device embolization and peri-device leak > 5 mm were rare in both groups and comparable, suggesting that operators adjusted for the different anatomy in patients with mitral valve disease. Postoperative hospitalization was longer in patients with MVD undergoing LAAO which reflects the higher burden of comorbidities such as chronic kidney disease (59.6% vs 36.8%), cardiomyopathy (21.2% vs 5.9%), and anemia (34.6% vs 21.6%).

Due to the proximity of the mitral valve to the orifice of the LAA, there is a concern that higher blood flow or regurgitant jet may impair endothelialization of the LAAO device once implanted [22]. Theoretically, a higher incidence of device-related thrombi (DRT) would be the result.

To our knowledge, there is no data on outcomes of patients with mitral valve disease after LAAO. Recently, a single-center case series of 122 AF patients undergoing TEER reported that around 50% would qualify for LAAO [9]. However, the study considered patients with a CHA2DS2-VASc score ≥ 3 and no contraindication to OAC as potential candidates. The recent European Society of Cardiology (ESC) guidelines on AF recommend LAAO only in patients with contraindication to OAC [4]. In our cohort, no differences in LA thrombi were observed; however, this may be due to low numbers of patients with MVD and even lower with complete echocardiographic follow-up data.

In addition, the impact of MVD on DRT is difficult to assess since larger LAA size itself is a strong predictor. For instance, in an analysis of 1739 patients from PREVAIL-AF, PROTECT-AF, and the continued registries, a 6% higher risk of DRT per mm LAA diameter increase (OR 1.06 per mm increase; 95% CI 1.01–1.12; *p* = 0.019) was observed [23]. A larger LAA diameter was also a predictor of DRT in the European EWOLUTION registry [24].

## Strengths and limitations

There are some caveats to be considered. No standardized process for patient selection, implanting procedure, or postprocedural management. Due to the observational study design, confounding factors cannot be excluded. Additionally, we were limited by the available data in the registry and had to rely on diameter for LA and LAA sizing without more sensitive volumetric values such as left atrial volume index. While our definition of patients with AF, MVD, and enlarged LA sizes probably reflects atrial myopathy, we cannot report sophisticated data such as magnetic resonance imaging or data from electroanatomic mapping.

Echocardiographic follow-up data are insufficient for conclusive incidences of DRT and peri-device leak. Adverse events were reported by the implanting center and maybe therefore unreliable. Also, this was a purely interventional study without any data on rhythm or rate control. Therefore, no association regarding AF treatment can be described in our registry. We also have limited data on the temporal relation of events, for instance, whether pericardial effusion was a result of transseptal puncture or device release.

Our study has some unique strengths as this is an industry-independent study under real-life conditions. This is also the very first work to systematically report the outcomes of patients with atrial myopathy due to mitral valve disease in LAAO. We can also report the 1-year mortality of this cohort. Additionally, this study uses different devices and employs a standardized 1-year follow-up independent of the implanting centers.

## Conclusion

Patients with MVD undergoing LAAO show atrial myopathy with larger LA diameter and LAA orifices. However, procedural success is not impaired and safety outcomes are comparable to patients without MVD. No differences in DRT or peri-device leak were observed and 1-year mortality is largely driven by comorbidities that must be addressed in management. Left atrial enlargement and MVD were not independent predictors of cardiovascular events.

## Supplementary Information

Below is the link to the electronic supplementary material.

Supplementary file1 (DOCX 23 kb)

## Abbreviations

AF: Atrial fibrillation
DRT: Device-related thrombus
LA: Left atrium
LAA: Left atrial appendage
LAAO: Left atrial appendage occlusion
MR: Mitral regurgitation
MVD: Mitral valve disease
NOAC: Non-vitamin K oral anticoagulants
OAC: Oral anticoagulants
SMVR: Surgical mitral valve repair
TEER: Transcatheter edge-to-edge repair
