# Supplementary material for: Association of atrial myopathy in mitral valve disease on safety outcomes in left atrial appendage closure
Source: Clin Res Cardiol. 2023 Feb 5;112(6):824–33. doi: 10.1007/s00392-022-02151-7 (PMC10241670; doi:10.1007/s00392-022-02151-7)
Supplement: Supplementary file 1 — Supplementary file1 (DOCX 23 kb) [file 392_2022_2151_MOESM1_ESM.docx]

**Supplement**

|  | **MVD cohort**  **(52)** | **No-MVD cohort**  **(476)** | **P-value** | **OR (95 %-CI)** |
| --- | --- | --- | --- | --- |
| **Moderate complications** |  |  |  |  |
| TIA | 0.0% (0) | 0.0% (0) | --- | --- |
| Non-fatal CPR | 0.0% (0) | 0.4% (2) | 1.00 | --- |
| Moderate bleeding | 0.0% (0) | 1.79% (9) | 1.00 | --- |
| Access site infection | 0.0% (0) | 0.20% (1) | 1.00 | --- |
| Groin hematoma | 1.92% (1) | 3.38% (17) | 1.00 | 0.56 (0.07-4.30) |
| Pericardial effusion – conservative treatment | 1.92% (1) | 1.79% (9) | 1.00 | 1.08 (0.13-8.67) |
| Hemo-/Pneumothorax – interventional treatment | 1.92% (1) | 0.0% (0) | 0.094 | --- |
| Hemo-/Pneumothorax – conservative treatment | 0.00 % (0) | 0.00 % (0/503) | --- | --- |
| Device embolisation – at index procedure | 1.92 % (1) | 0.99 % (5/503) | 0.45 | 1.95 (0.22-17.04) |
| **Overall complications (severe & moderate)** | 19.2 % (10) | 12.5 % (63/503) | 0.19 | 1.66 (0.79-3.48) |

**Table 1: Further In-hospital safety data:** MVD: mitral valve disease; OR: odds ratio; CI: confidence interval; transient ischemic attack; CPR: cardiopulmonary resuscitation; displayed are percentages and numbers or median and quartiles; P-values <0.05 are considered significant, tested with tested with Fisher`s exact test

|  | **MVD cohort**  **(52)** | **No-MVD cohort**  **(476)** | **P-value** |
| --- | --- | --- | --- |
| **Therapy at discharge** | **n=52** | **n=474** |  |
| Anticoagulation | 0.0% (0) | 3.4% (16) | 0.18 |
| DAPT | 92.3 % (48) | 84.8 % (402) | 0.14 |
| SAPT | 0.0 % (0) | 3.2 % (15) | 0.19 |
| Double antithrombotic therapy | 5.8 % (3) | 5.7 % (27) | 0.98 |
| Triple antithrombotic therapy | 1.9 % (1) | 2.5 % (12) | 0.79 |
| No antithrombotic therapy | 0.0 % (0) | 0.4 % (2 | 0.64 |
| PPI | 53.8 % (28) | 46.8 % (221) | 0.34 |
| NSAID | 19.2 % (10) | 2.5 % (12) | < 0.001 |
| **Therapy at one-year FU** | **n=35** | **n=387** |  |
| Anticoagulation | 8.6 % (3) | 4.9 % (19) | 0.35 |
| DAPT | 14.3 % (5) | 5.4 % (21) | 0.037 |
| SAPT | 68.6 % (24) | 77.3 % (299) | 0.25 |
| Double antithrombotic therapy | 2.9 % (1) | 1.0 % (4) | 0.34 |
| Triple antithrombotic therapy | 2.9 % (1) | 0.3 % (1) | 0.032 |
| No antithrombotic therapy | 0.0 % (0) | 0.0 % (0) |  |
| PPI | 51.4 % (18) | 40.1 % (155) | 0.19 |
| NSAID | 11.4 % (4) | 2.6 % (10) | 0.005 |

**Table 2: Antithrombotic therapy:** OR: odds ratio; CI: confidence interval; DAPT: dual antiplatelet therapy; SAPT: single antiplatelet therapy; PPI: proton pump inhibitor; NSAID: non-steroidal anti-inflammatory drugs; displayed are percentages and numbers; P-values <0.05 are considered significant, tested with either Pearson chi-squared test or Mann-Whitney-Wilcoxon test
